# Mesenteric cysts in naevoid basal cell carcinoma syndrome: a mimic of metastatic disease

**DOI:** 10.1111/bjd.14224

**Published:** 2015-12-14

**Authors:** N. Rajan, S. Brown, S. Ward, P. Hainsworth, P. Hodgkinson, P. Pieniazek, A. Husain, R. Plummer

**Affiliations:** ^1^ Institute of Genetic MedicineNewcastle UniversityNewcastle upon TyneNE1 3BZU.K.; ^2^ Department of DermatologyRoyal Victoria InfirmaryNewcastle upon TyneU.K.; ^3^ Department of SurgeryFreeman HospitalNewcastle upon TyneU.K.; ^4^ Department of RadiologyRoyal Victoria InfirmaryNewcastle upon TyneU.K.; ^5^ Department of HistopathologyRoyal Victoria InfirmaryNewcastle upon TyneU.K.; ^6^ Northern Centre for Cancer CareFreeman HospitalNewcastle upon TyneU.K.

dear editor, Novel treatments that target the oncogenic hedgehog signalling pathway are revolutionizing the treatment of cutaneous basal cell carcinoma (BCC) in patients with naevoid basal cell carcinoma syndrome (NBCCS). Computed tomography (CT) imaging screening examinations are included at baseline in clinical trials of these agents, to establish the absence of metastatic disease. These may reveal hidden, asymptomatic features of this syndrome that can mimic metastatic disease, such as mesenteric cysts as described in this report.

**Figure 1:**
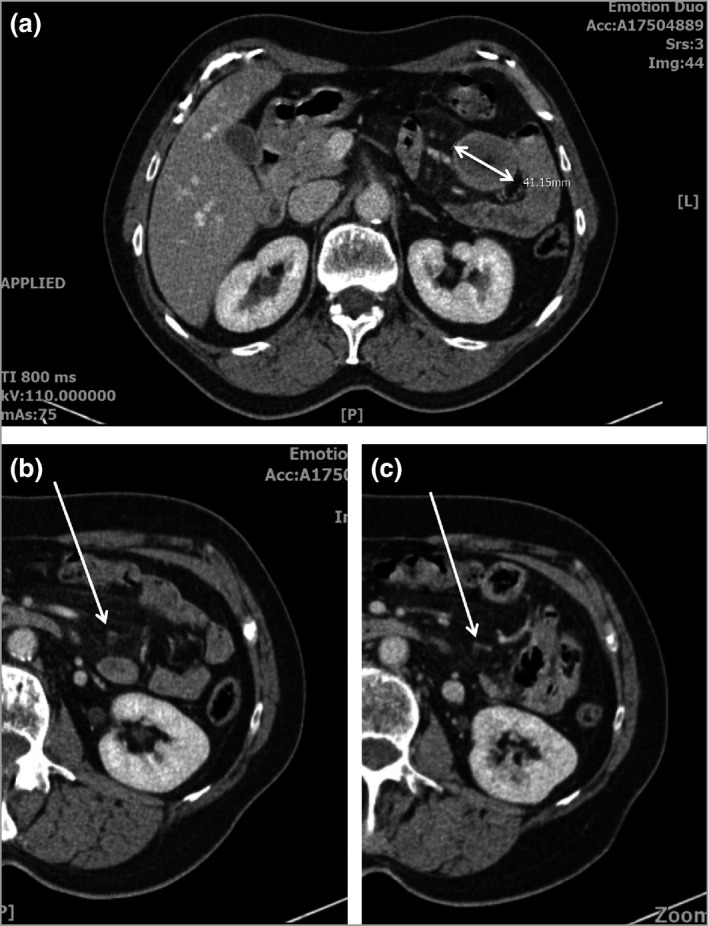
(a) Baseline pretreatment computed tomography (CT) image highlighting a large mesenteric cyst: the double‐headed white arrow indicates a span of 41·15 mm width. (b) A small mesenteric cyst detected at baseline CT before treatment, indicated with a white arrow. (c) Two months after commencing vismodegib treatment, the same mesenteric cyst is smaller.

A 61‐year‐old woman with NBCCS was enrolled in a clinical trial of a systemic smoothened inhibitor (vismodegib). The patient had a history of jaw cysts requiring surgical excision in her teens, and excision of BCCs from her fourth decade. She had previously received multiple treatments to manage BCCs, including topical 5‐fluorouracil, photodynamic therapy, cryotherapy, curettage and excision. Clinical examination revealed hypertelorism, macrocephaly and multiple BCCs. Genetic testing demonstrated a germline, heterozygous five‐nucleotide deletion in exon 3 (c.456_460del) of the patched 1 gene (*PTCH1*) that disrupted the reading frame, resulting in a premature termination codon. As part of the clinical trial protocol, she underwent CT imaging of her chest, abdomen and pelvis. This revealed multiple mesenteric lesions, the largest of which measured 4·1 cm in diameter (Fig. 1a). The differential diagnoses included mesenteric cysts and metastatic disease. The patient proceeded to receive vismodegib on the basis that the findings were likely to represent cystic disease.

**Figure 2:**
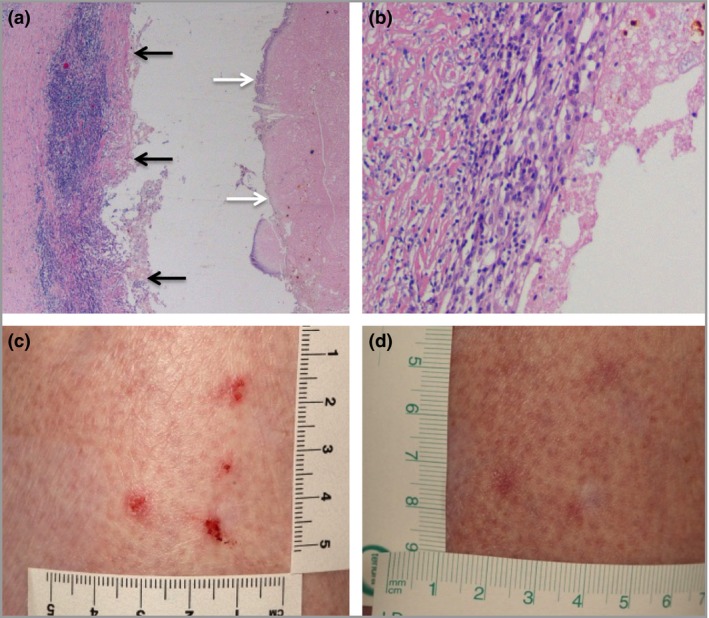
(a) Histology of the cyst revealed central, amorphous, necrotic areas (white arrows) lined with dense fibrocollagenous tissue and chronic inflammation (black arrows), separated by an artefactual cleft. No evidence of neoplasia was noted (haematoxylin and eosin, original magnification ×20). (b) A high‐power view of the cyst wall in (a) demonstrating a lymphocytic infiltrate (original magnification ×40). (c) Multiple superficial basal cell carcinomas seen on the leg before treatment with vismodegib and (d) at 8 months post‐treatment at the same site.

A repeat CT after a 2‐month interval demonstrated that the largest cyst had not grown, and some smaller cysts were reducing in size (Fig. 1b, c). However, the patient elected to undergo laparoscopic biopsy, to exclude the remote possibility of metastatic disease. This revealed the presence of multiple nodules within the mesentery of the small bowel, of which the largest lesion was excised. Histopathological examination revealed central necrotic areas lined with dense fibrocollagenous tissue, suggestive of a chronic process; there was no evidence of malignancy (Fig. 2a, b). A further abdominal CT scan at 6 months demonstrated the absence of remaining small cysts. Eight months after the start of treatment the majority of the BCCs had resolved and the requirement for cutaneous surgical intervention was reduced (Fig. 2c, d).

Inhibiting the hedgehog signalling pathway in patients with NBCCS is a recent treatment strategy for BCC.^1^ Patients with NBCCS frequently carry heterozygous germline mutations in *PTCH1*,^2^ a gene that encodes a transmembrane glycoprotein, which is crucial for the regulation of hedgehog signalling in cells. The type of mutation does not appear to influence the severity of the phenotype. Loss of function of the PTCH1 protein in BCC cells is thought to be the key driver that underlies BCC formation. As PTCH1 is required for negative regulation of smoothened, another protein in the hedgehog signalling pathway, aberrant oncogenic hedgehog signalling ensues, associated with BCC growth. In keeping with this model, a landmark study by Tang *et al*.^1^ demonstrated that vismodegib, a molecule that can compensate for loss of PTCH1 by inhibiting smoothened activity, both reduced BCC burden and inhibited growth of new BCCs.

Asymptomatic features of NBCCS, such as mesenteric cysts in our patient, are likely to be detected as therapies such as vismodegib are assessed in clinical trials and patients undergo associated baseline imaging investigations. Mesenteric cysts, rare in the general population, are recognized as a minor clinical diagnostic criterion in NBCCS.^3^ In the absence of prospective studies where CT imaging is available from cohorts of *PTCH1* mutation carriers, the true frequency of mesenteric cysts in NBCCS is unknown. Mesenteric cysts in NBCCS are usually incidental findings on exploratory laparotomy.^4^ In a series of 36 patients with NBCCS, three cases of mesenteric cysts were identified at exploratory laparotomy.^4^ Uncommonly, patients may experience symptoms, such as colicky abdominal pain.^5^ Mesenteric cysts in NBCCS are thin walled and can range from 2 to 14 cm in diameter. The content of mesenteric cysts is often chylous but can also be haemorrhagic.^6^ Histological examination of mesenteric cysts demonstrates a lining consisting of fibrous tissue and smooth muscle, often surrounded by a layer of lymphatic cells.

Mesenteric cysts and metastatic disease can be difficult to distinguish radiologically. Serial CT imaging allows assessment of increase in lesion size suggestive of metastatic disease over benign cysts. Fludeoxyglucose‐labelled positron emission tomography scans also show lack of uptake in these cysts, which may serve as a useful adjunct in case of uncertainty.^7^ Notably, in our case smaller mesenteric cysts (Fig. 1b) demonstrated a reduction in size after 2 months of vismodegib treatment (Fig. 1c). This finding has not been previously reported to our knowledge. Furthermore, there was an absence of small cysts at 6 months as assessed by CT imaging. While this preliminary finding awaits confirmation in future studies, it may suggest that mesenteric cyst maintenance in NBCCS is dependent on aberrant hedgehog signalling, which is amenable to intervention by smoothened inhibitors.
